# A College of Nursing

**Published:** 1916-01-15

**Authors:** A. Stanley

**Affiliations:** Chairman, Joint War Committee, British Red Cross Society and Order of St. John. 83 Pall Mall.


### THE EDITOR'S LETTER-BOX.

[THE letter we publish on this page was received too late for publication in our last issue. It has been sent to, and is at present under the considera tion of, the nurse-training schools. It has excited considerable interest and has already been received with favour.—ED. THE HOSPITAL.]

### A COLLEGE OF NURSING.

BY THE HONBLE. ARTHUR STANLEY, M.P., M.V.O.

SIR,—During my past year's work as Chairman of the Joint War Committee of the British Red Cross Society and Order of St. John I have been struck by the total lack of organisation amongst the various authorities responsible for the training of nurses and by the need for organisation amongst the nurses themselves. Occupying a neutral position in relation to these pressing problems I have given much time and attention to considering how, in the interests of the public and of the nurses themselves, the present unsatisfactory condition of affairs may be remedied.

### A CALL FOR UNITY.

For something like twenty-five years there has been more or less active agitation in favour of the registration of trained nurses, but for causes into which it is unnecessary here to enter, this movement and others of a similar nature have hitherto failed to attain the object sought.

There is no unanimous feeling either amongst those responsible for the training of nurses or amongst nurses themselves in favour of any system of State registration. Nevertheless, I am convinced that something should be done at once to co-ordinate the various interests involved, and, without prejudice to ultimate developments, whether by legislation or otherwise, my own view is that for the time at least we must rely upon a voluntary scheme of co-operation amongst the nurse-training schools throughout the country.

I have confidence that a representative association would speedily attract so much support from the nursing profession as to lend great weight and authority to any decisions at which it might arrive.

### A PURELY VOLUNTARY BODY.

Just as the Royal Colleges of Physicians and Surgeons through the Conjoint Board organise the teaching and examination of medical students, as the chartered institutes of accountants, of surveyors, engineers, and other bodies, as barristers and solicitors organise the teaching and examining of candidates for entrance to their respective professions, so do I feel most strongly that now is the right time for some such movement in the nursing profession.

With this end in view it is suggested that a College of Nursing shall be founded. This college shall be a purely voluntary body, which will aim at securing the support and sympathy of the governors of hospitals to which nurse-training schools are attached, of the leading members of the medical profession, of the matrons and lecturers at these nurse-training schools, and last, but not least, of the trained nurses themselves.

### COMPLETE REPRESENTATION AIMED AT.

I suggest that promoters of the college should be sought amongst the chairmen and governors of leading hospitals, physicians and surgeons lecturing to nurses, the principals of nurse-training schools and of nursing associations, and other persons interested in the education of women, and I hope your hospital may be willing in due time to nominate representatives to act in this capacity. The promoters, having obtained the sanction of the Board of Trade to the registration of the college with its memorandum and articles of association, should appoint the first council of management, two-thirds of whom should be matrons of hospitals or superintendents of nursing. For effective administration it is essential that the council of management should be relatively small in numbers, but in order to secure a proper representation of all interests it is proposed that the council should form a large consultative board drawn from all classes of nurse-training schools and nursing associations, and from nurses in practice throughout the country. Further, it is suggested that the council should always invite and receive a report from the consultative board before coming to a determination either upon courses of study and technical training for persons intended for the nursing profession or upon the conditions under which recognition may be extended to nursing schools. The council should also form an examination board, which will advise upon the appointment of examiners, the scheme for examinations, and the acceptance under safeguards of internal examinations in recognised training-schools to qualify for the certificate of proficiency in nursing to be granted by the college.

In addition to examining and giving qualifications to nurses, the college should take power to exercise similar functions in all branches of women's work connected with hospitals, whether naval, military, or civil, and to give certificates of proficiency to those who pass the necessary examinations.

### CONSTRUCTIVE CRITICISM INVITED.

Such are the broad outlines of the scheme, and I should be glad to know whether they meet with the general approval of your hospital committee. It is put forward with the idea of securing greater uniformity of training and curriculum for nurses, fuller co-operation between the nursing schools, and the better organisation of the nursing profession generally. I shall be glad to submit to the advisers who have already helped me any criticism you may be pleased to make. I have reason to hope that, should these proposals meet with sympathetic support, I may be able to secure financial assistance to launch the college in a suitable building, but before asking the Board of Trade's approval to omit the word "limited" from the title of the association I must be assured of a substantial backing throughout the country. When this step is gained I shall have the pleasure of asking your committee to nominate your representative to take part in the establishment and development of the college, which may, I hope, promote the welfare of trained nurses and of all other women engaged in attendance upon the sick and wounded, and may thus do lasting service to our nation and country.—Yours faithfully,

A. STANLEY,

Chairman, Joint War Committee, British Red Cross Society and Order of St. John.

83 Pall Mall.

December 30, 1915.

